# APOPHYSEAL FRACTURE OR AVULSION OF THE GREATER TROCHANTER

**DOI:** 10.1590/1413-785220162403155803

**Published:** 2016

**Authors:** ANDERSON FREITAS, SÍLVIO LEITE DE MACEDO

**Affiliations:** 1. Hospital Ortopédico e Medicina Especializada (HOME-DF), Hip Surgery Training Service, Brasília, DF, Brazil.

**Keywords:** Hip fractures, Adolescent, Osteonecrosis.

## Abstract

The apophyseal fracture or avulsion of the greater trochanter occurs in skeletally immature patients. It has at etiological factors indirect mechanisms (avulsion) and direct mechanisms (trauma on the trochanter), the latter being the most common. The clinical presentation is easily identified and a simple X- ray can confirm the diagnosis. Regardless of the treatment chosen, this pathology has a high correlation with osteonecrosis of the femoral head, even as a rare fact, however with innocent appearance, on this pathology.

## INTRODUCTION

The prevalence of fractures of the proximal femoral end in children represents less than 1% of hip fractures in adults.^1^ ^,^ ^2^ The physeal injuries of the proximal femur represent only 0.1% of physeal fractures reported in Olmstead County, Minnesota, US, in the 80's.^3^

Fractures of the proximal femur in immature skeletons, most commonly associated to osteonecrosis of the femoral head are Delbert's type I and II femoral neck fractures.^4^ ^,^ ^5^

The vascularity of the proximal femoral epiphysis reported by Trueta,^6^ Chung^7^ and Ogden^8^ describes the proximity of vascular structures to the trochanteric region. Fractures that compromise the integrity of the circumflex medial artery and its cervical branches have a great chance to evolve with osteonecrosis of the femoral head, either by direct damage to the vascular structures or by intracapsular compression hematoma.^6^ ^-^ ^8^

However, despite its very low occurrence and few reports in the literature, the apophyseal fracture or avulsion of the greater trochanter has a high incidence of osteonecrosis of the femoral head.^9^ ^,^ ^10^

Therefore, due to its rarity and appearance of an innocent fracture, with nonetheless catastrophic evolution, this study has the goal to contribute to the literature, updating information on this condition in order to prevent that misinformation may lead to worsening of its progression.

## LITERATURE REVIEW

Initially described by Poland in 1898,^11^ the apophyseal traumatic detachment of the greater trochanter presented a terrible evolution on the twelve cases followed by the author, of which only two could be diagnosed in life, all other cases evolved to death from sepsis. We believe that such development was due to the high risk of infection of the fracture hematoma associated with the region involved, being the proximal femur a highly vascularized area with many cancellous bone, and leading to quickly dissemination of the hematoma infection through the bloodstream, causing sepsis and death.

Thienhaus in 1904^12^ reported a case of a fracture of the apophysis of the greater trochanter in an eleven-year-old victim of fall. The author described his concern to differentiate large deviation cases, because he believed that such cases were more likely to evolve to death by sepsis, due to the large hematoma. He added that in such cases the best approach should be surgical, in order to drain the hematoma, suture the abductor complex and implant a drain.

Linhart et al. in 1984^13^ reported the occurrence of necrosis of the femoral head in a twelve-year-old patient after a fracture with involvement of large and small trochanter, describing that factors related to vascular anatomy of the proximal femur and intimacy with the trochanteric region could be related to the traumatic injury of the femoral head vascularity.

Kaweblum et al. in 1993^9^ were the first authors to describe an apophyseal detachment of the greater trochanter alone in a twelve-year-old patient after fall from height, treated with surgery with open reduction and internal fixation, who evolved twelve months after surgery with avascular necrosis of the femoral head and major mobility limitation of the affected hip. This paper reported the possibility of necrosis by iatrogenic injury of the femoral head vascularity.

O'Rourke et al. in 2003,^10^ in an excellent article reporting two cases of apophyseal detachment of the isolated greater trochanter with little deviation treated conservatively and another case with large deviation, treated with minimally invasive internal fixation with percutaneous screws, described a severe course with osteonecrosis of the femoral head in both cases. Those authors were the first to draw attention to the anterior deviation of the fractured apophyseal fragment and strengthened the correlation of vascular injury at the time of trauma, since it presented a slightly diverted case treated conservatively with the same evolution as surgery. The authors also mentioned that such a shift in young patients, unlike the isolated fractures of the greater trochanter in adults, was due to the rupture of the external rotators of the hip, due to bone immaturity. Moreover, there is a high possibility of traumatic rupture being the cause of proximal femoral vascularity.

### Clinical Presentation

Clinical assessment and trauma history allow a high degree of diagnostic suspicion. The patient has pain in the anatomical topography of the greater trochanter that worsens with palpation and may present difficulty or inability to walk. An exploration maneuver of the abductor mechanism of the hip, as Trendelenburg's, can exacerbate the symptoms or be impractical, depending on the degree of injury. The mechanism of injury may be related to direct trauma in the region (most common) or a great effort in games or sports practices with subsequent pain (indirect trauma mechanism or avulsion).

It is important to mention that historical reports have described infection after trauma in this region. Therefore, trauma history in this region that evolves with signs of redness, fever and pain, should be evaluated for this complication.

### Diagnostic Imaging

**Figure 1:**
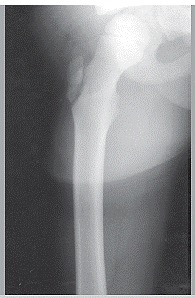
Anterior posterior radiograph of the right hip showing a fracture or avulsion of the apophysis of the greater trochanter with a slight deviation to the top.

**Figure 2:**
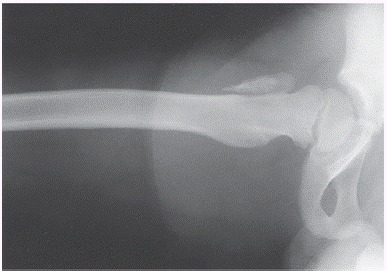
Radiography in frog position of the right hip, with anterior fracture dislocation or avulsion of the apophysis of the greater trochanter.

A simple pelvic radiograph with the hip in neutral and double abduction (frog) position may elucidate the diagnosis. In cases a deviation (mild or severe) of the apophysis of the greater trochanter in the neutral position is noticed (Figure 1), accompanied by an anterior shift at the frog position (Figure 2), a better imaging evaluation can be performed with using CT scan. However, for very young patients with immature reproductive structures, it is wise to employ CT scans when diagnosis with simple X-rays is not possible, or in cases that may be associated with other fractures (pelvic or femoral neck fracture).

## TREATMENT

Because it is a rare occurrence, there is no consensus on the best treatment. Therefore, three different ways of treatment are described in the literature, namely: conservative treatment, open reduction and internal fixation and closed reduction with minimally invasive internal fixation. All of them evolved with the same results, i.e., osteonecrosis of the femoral head.

The description of open reduction and internal fixation was performed by Kaweblum et al. ^9^ in a deviated fracture of a twelve-year-old patient, using two nonparallel cancellous screws with a double-abduction apparatus for three weeks without ambulation. After this period, normal painless mobility was observed, and ambulation with load less crutches was allowed. After three months of evolution, the patient developed painful hip mobility and difficulty to walk. X-ray showed a collapse of the femoral head and lateral subluxation of the hip, compatible with osteonecrosis of the femoral head.

Description of conservative treatment with closed reduction and internal fixation are part of the same paper by O'Rourke and Weinstein,^10^ a technique applied to a five-year-old patient with history of pain in the greater trochanter after trampling, treated with bed rest for three weeks followed by three weeks of load less crutch-assisted walking. After this period, due to a good tolerance to pain and good mobility, patient was released to the usual activities. Six months later, patient evolved with pain and low mobilization of the affected hip, x-ray showed complete collapse of the femoral head. (Figures 1, ^2^ and ^3^) The closed reduction and internal fixation case occurred in a thirteen-year-old patient, who described pain in the greater trochanter after effort during a football game, without direct trauma. X-ray showed a large deviation from the apophysis of the greater trochanter, which was treated with closed reduction and internal 

**Figure 3:**
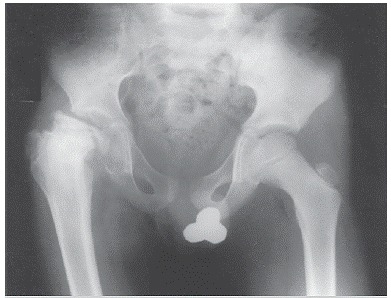
Anterior posterior radiograph of the pelvis, ato ne year evolution after trauma, showing a major colapse and subluxation of the femoral head to the right, characteristic of osteonecrosis of the femoral head.

fixation with two cannulated screws. Ambulation occurred with crutches for six weeks and after evaluation of mobility and pain, patient was released to daily activities. Screws were removed with five months postoperatively and at this time the patient even participated to basketball games. Eighth month later pain and difficulty of mobilizing in the operated hip started. X-ray showed collapse of the femoral head, typical for oste onecrosis.

### Differential Diagnosis 

The isolated fracture of the apophysis of the greater trochanter presents difficulties to be diagnosed. However, less classical presentation where x-ray does not show fractures, other intra-and extra-joint conditions must always be considered in adolescents, such as femoral neck fracture, pelvic avulsion, hip synovitis, and septic arthritis.

## CONCLUDING REMARKS

The fracture or avulsion of the greater trochanter apophysis is a rare traumatic condition, which appears to be harmless. It has, however, been documented in the literature, to the best of the authors' knowledge, as a serious and limiting condition, with osteonecrosis of the femoral head. When we encounter this diagnosis, we must inform the patients and their families on the possibility of serious complications, whichever treatment is adopted. We believe that the infection described earlier regarding this condition is no longer a serious problem if handled correctly, since this complication has been described by older publications, a time with limited or no use of antimicrobials, and have been cited in this review only for historical reasons.

## References

[1] Canale ST, Bourland WL (1977). Fracture of the neck and intertrochanteric region of the femur in children. J Bone Joint Surg Am.

[2] Morrissy RT (1984). Fractured hip in childhood. Instr Course Lect.

[3] Peterson HA, Madhok R, Benson JT, Ilstrup DM, Melton 3rd LJ (1994). Physeal fractures Part 1. Epidemiology in Olmsted County, Minnesota, 1979-1988. J Pediatr Orthop.

[4] Davison BL, Weinstein SL (1992). Hip fractures in children a long-term follow-up study. J Pediatr Orthop.

[5] Colonna PC (1928). Fracture of the neck of the femur in childhood a report of six cases. Ann Surg.

[6] Trueta J (1957). The normal vascular anatomy of the human femoral head during growth. J Bone Joint Surg Br.

[7] Chung SM (1976). The arterial supply of the developing proximal end of the human femur. J Bone Joint Surg Am.

[8] Ogden JA (1974). Changing patterns of proximal femoral vascularity. J Bone Joint Surg Am.

[9] Kaweblum M, Lehman WB, Grant AD, Strongwater A (1993). Avascular necrosis of the femoral head as sequela of fracture of the greater trochanter A case report and review of the literature. Clin Orthop Relat Res.

[10] O&apos;Rourke MR, Weinstein SL (2003). Osteonecrosis following isolated avulsion fracture of the greater trochanter in children A report of two cases. J Bone Joint Surg Am.

[11] Poland J (1898). Traumatic separation of the epiphyses.

[12] Thienhaus CO (1906). XI Epiphyseal Separation of the Great Trochanter With Report of a Case. Ann Surg.

[13] Linhart W, Stampfel O, Ritter G (1984). Post-traumatic femur head necrosis following trochanter fracture. Z Orthop Ihre Grenzgeb.

